# Echocardiography for Volume Assessment in Acute Myocardial Infarction

**DOI:** 10.7759/cureus.47946

**Published:** 2023-10-30

**Authors:** Satish Ramteke, Vinit Kumar, Dhananjay Kumar, Manish Gupta

**Affiliations:** 1 Department of Cardiology, Noble Multispeciality Hospital, Bhopal, IND; 2 Department of Cardiology, Laxmipat Singhania (LPS) Institute of Cardiology and Cardiac Surgery, Kanpur, IND; 3 Department of Cardiology, Narayan Medical College and Hospital, Rohtas, IND

**Keywords:** echocardiography, prognosis, inferior vena cava, heart failure, blood volume

## Abstract

Background

Errors caused by improper volume estimation increase acute mortality rates in acute myocardial infarction (AMI). We aimed to determine volume status in AMI patients using echocardiography and to correlate the findings with clinical outcomes.

Methods

This cross-sectional, single-center study was performed at a tertiary care center in India between August 2017 and September 2020 involving AMI patients. We performed echocardiography for all patients. Parameters such as left ventricle (LV) and atrium size, LV end-diastolic pressure, inferior vena cava (IVC) size and size variation, velocity stroke volume, and velocity time integral variation were measured. B-lines were recorded by scanning 32 regions on the anterior chest in the supine position using cardiac probes of echocardiography.

Results

A total of 184 patients were enrolled in the study with male predominance (82.1%). The mean age of patients was 58.2 ± 10.7 years. Dilated (>2.1 cm) and collapsible (<50%) IVC, and B-lines were significantly associated with heart failure (HF) (p<0.001; r=0.87 and p<0.001; r=0.74, respectively). The area under receiver operating characteristics (AUROC) curve to diagnose HF at a cut-off value of >10 for B-lines was 0.897 (0.842-0.951). AUROC curve for IVC size in diagnosing hypovolemia was 0.063 (0.000-0.130).

Conclusions

Volume status based on IVC size and B-lines detected by echocardiography has a strong prognostic value in AMI patients and should be included in the routine assessment of these patients.

## Introduction

Hemodynamic evaluation is essential for diagnosis and decision-making regarding treatment strategies in patients with myocardial infarction (MI) [1]. Echocardiography can detect regional wall motion and hemodynamic abnormalities, and changes in myocardial thickness, among other uses [1]. Lung ultrasound detects pulmonary fluid by the identification of the presence of sonographic artifacts, known as B-lines, which suggest thickened interstitia or fluid-filled alveoli [2]. B-lines, usually seen in patients with congestive heart failure (HF), correlate with elevated pulmonary capillary wedge pressure and pulmonary fluid. B-lines demonstrate high sensitivity and specificity for alveolar interstitial fluid. Another popular predictor of volume status is the inferior vena cava (IVC) diameter. Studies have demonstrated that dilated IVC at admission is associated with a poor prognosis of HF [3,4].

According to the European Society of Cardiology (ESC) 2016 guidelines for the diagnosis and treatment of acute and chronic HF, thoracic ultrasound may be considered for confirmation of pulmonary congestion in patients with acute HF, with class IIb recommendation [5]. Similarly, ultrasound measurement of IVC may be considered for the assessment of volume status in patients with HF with the same recommendation [5]. However, there is a scarcity in the literature on the assessment of volume status among patients with AMI.

Evaluation of systemic congestion, along with the assessment of low cardiac output, may be beneficial in identifying acute MI (AMI) patients at a higher risk of poor prognosis, making it useful in deciding the appropriate treatment [6]. The current study aimed to investigate the volume status of patients with AMI and to correlate clinical and echocardiographic parameters of volume status.

## Materials and methods

Study design and population

Between August 2017 and September 2020, a cross-sectional, single-center study was performed at a tertiary care center in India. The study was approved by the institutional ethics committee (No.: RKC/Ethics/01; approved on 08 May 2017) and was conducted per the Declaration of Helsinki (as revised in 2013). Written informed consent was obtained from all individual participants.

HF patients with preserved ejection fraction (EF) (≥50%), reduced EF (<50%), and clinical HF patients as per Framingham criteria were included [7]. Patients with third and fourth stages of chronic kidney disease and end-stage renal disease were excluded.

Clinical examination and echocardiography

We clinically examined patients for signs of hyper- or hypovolemia. Echocardiography was performed using the Philips iE33 echo system (Philips, Amsterdam, Netherlands) at the time of admission to measure parameters such as left ventricle (LV) and left atrium size, LV end-diastolic pressure, IVC size, and IVC size variation, right atrial pressure, passive leg raising stroke volume, IVC dispensability index, aortic peak velocity, and velocity time integral variation. B-lines were recorded by scanning 32 regions on the anterior chest in the supine position using cardiac probes of echocardiography.

Data analysis

The sample size was determined by using the sample size calculation formula for cross-sectional studies: N = ((Z_1_ - α/2)^2^ × p × q)/L^2^, where the value of Z_1_ - α/2 =1.96 considering 95% confidence level, P = proportion of cases with reference (pilot) study, q = (100-p) = 100-20 = 80, and L = precision in absolute term was considered as 6. A pilot study was carried out taking 10 patients with AMI; two of them showed B-lines; therefore, p = proportion of cases that showed B-lines in echocardiography was taken as 20%.

Sample Size Calculation

 N=3.84×20×80/36=170.66

The continuous and categorical variables were presented as mean ± SD and frequencies (percentage), respectively. The Chi-squared test, Student's t-test, and Mann-Whitney U test were performed as applicable. A p-value <0.05 was considered significant. Statistical Package for Social Sciences version 20.0 (IBM Inc., Armonk, New York) was used for statistical analyses.

## Results

This cross-sectional study involved 184 AMI patients. There were 151 (82.1%) males. The mean age of patients was 58.2 ± 10.7 years, and 39.7% of patients were between 56-65 years. Clinical investigations are given in Table 1.

**Table 1:** Patient characteristics, clinical features, and patient distribution according to clinical investigations and parameters suggestive of hypovolemia Data are expressed as n (%) AHF - acute heart failure; BNP - B-type natriuretic peptide; EF - ejection fraction; HF - heart failure; IVC - inferior vena cava; JVP - jugular venous pressure; LAD - left anterior descending artery; LV - left ventricle; LVIDd - left ventricular internal diameter end diastole; NT-proBNP - N-terminal pro B-type natriuretic peptide; RCA - right coronary artery; RVIDd - right ventricular internal dimension end diastole; SBP - systolic blood pressure.

Patient characteristics (N=184)			n (%)
Age			
≤45			23 (12.5)
46-55			49 (26.6)
56-65			73 (39.7)
66-75			25 (13.6)
>75			14 (7.6)
Male			151 (82.1)
Clinical features			
Symptoms	Paroxysmal nocturnal dyspnea		57 (31.0)
	Orthopnea		82 (44.6)
	Shortness of breath		123 (66.8)
	Chest pain		172 (93.5)
	Orthostatic symptom		25 (13.6)
Signs	JVP	<3 cm	104 (56.5)
		>3 cm	80 (43.5)
	Pulse rate	<60	6 (3.3)
		61-100	109 (59.2)
		>100	69 (37.5)
	Pulse type	Normovolemic	96 (52.2)
		Hypovolemic	88 (47.8)
	SBP	<100 mmHg	85 (46.2)
		>100 mmHg	99 (53.8)
	Crepitation	Bilateral basal 1/3	14 (7.6)
		Bilateral full lung field	14 (7.6)
		Up to 1/2 of lung field	30 (16.3)
		None	126 (68.5)
	S3		49 (26.6)
	S4		42 (22.8)
	Shrunken eyes		25 (13.6)
	Dryness of tongue		25 (13.6)
	Dry skin		25 (13.6)
	Postural hypotension		25 (13.6)
Complications	With reduced EF (<50%)	AHF	28 (15.2)
		Chronic HF	30 (16.3)
		AHF with pulmonary edema	6 (3.3)
		AHF with cardiogenic shock	6 (3.3)
	With preserved EF (≥50%)	AHF	10 (5.4)
		Chronic HF	0
		AHF with pulmonary edema	0
		AHF with cardiogenic shock	0
	No HF		104 (56.5)
Investigations			
LVIDd	Small (<42 in males; <38 in females)		12 (6.5)
	Normal (42-58 in males; 38-52 in females)		154 (83.7)
	Dilated (>58 in males; >52 in females)		18 (9.8)
LV contractility	Global hypokinesia		32 (17.4)
	Normal		12 (6.5)
	Reduce contractility in RCA territory		40 (21.8)
	Reduce contractility in LAD territory		100 (54.3)
IVC size	Small (<1.2)		16 (8.7)
	Normal (1.2-2.1)		88 (47.8)
	Dilated (>2.1)		80 (43.5)
IVC collapsibility	<50%		80 (43.5)
	>50%		104 (56.5)
RVIDd	Small (<19)		21 (11.4)
	Normal (19-35)		119 (64.7)
	Dilated (>35)		44 (23.9)
Chest X-ray	B/L scattered infiltration		5 (2.7)
	Infiltration 2/3 of lung field		30 (16.3)
	Normal		126 (68.5)
	Perihilar infiltration		21 (11.4)
	Pleural effusion		2 (1.1)
B-lines	<10		96 (52.2)
	>10		88 (47.8)
BNP	<100		91 (49.5)
	>100		93 (50.5)
NT-proBNP	<900		99 (53.8)
	>900		85 (46.2)
Mitral E/e’	<15		109 (59.2)
	>15		75 (40.8)
Tricuspid E/e’	<6		104 (56.5)
	>6		80 (43.5)
Pericardial effusion	Present		52 (28.2)
	Absent		132 (71.7)
Parameters suggestive of hypovolemia			
Orthostatic symptoms			25 (13.6)
Dryness of tongue			25 (13.6)
Shrunken eyes			25 (13.6)
Dry skin			25 (13.6)
Postural hypotension			25 (13.6)
Tachycardia			69 (37.5)
Hypovolemic pulse			88 (47.8)
SBP <100mmHg			85 (46.2)
JVP <3 cm			104 (56.5)
IVC <1.2 cm with collapsibility >50%			16 (8.7)
IVC >2 cm with collapsibility <50%			80 (43.5)
Paroxysmal nocturnal dyspnea			57 (31.0)
Orthopnea			82 (44.6)
JVP >3 cm			80 (43.5)
S3			49 (26.6)
S4			42 (22.8)
Crepitation			58 (31.5)
Mitral E/e' increased (>15)			75 (40.8)
Tricuspid E/e' increased (>6)			80 (43.5)
B-lines >10			88 (47.8)
Chest x-ray infiltration			58 (31.5)
BNP increased (>100)			93 (50.5)
NT-proBNP increased (>900)			85 (46.2)
Pericardial effusion			62 (28.2)

The majority of patients were diagnosed with anterior wall MI (55.4%), followed by inferior wall MI (21.7%). A total of 80 patients (43.5%) presented with HF, of which 38 (20.7%) had acute HF (AHF) and 30 (16.3%) had chronic HF. Reduced EF (<50%) was observed in 15.2% of AHF patients. All patients with chronic HF had reduced EF, and all those with pulmonary edema and cardiogenic shock presented with AHF.

Small and collapsible IVC (>50%) was observed in 84.2% of patients with hypovolemia, whereas dilated and collapsible IVC (<50%) was observed in 100% of patients with hypervolemia. The association between IVC size and volume status was significant (P<0.001). Table 1 also depicts patient distribution according to parameters suggestive of hypovolemia. Dilated (>2.1 cm) and collapsible (<50%) IVC was significantly associated with HF (p<0.001). As hypervolemia progressed, IVC became dilated and collapsible (correlation coefficient 0.869; p<0.001). Table 2 describes the association of IVC size and collapsibility with various parameters.

**Table 2:** Association of IVC size and collapsibility with various parameters Data are expressed as n (%) IVC - inferior vena cava; SBP - systolic blood pressure; JVP - jugular venous pressure; HF - heart failure; AHF - acute heart failure EF - ejection fraction

Parameters of hypovolemia		IVC			p-value
		Small and collapsibility >50% (n=16)	Normal and collapsibility >50% (n=88)	Dilated and collapsibility <50% (n=80)	
Orthostatic symptoms	Yes	16 (100)	3 (3.4)	6 (7.5)	0.001
	No	0 (0)	85 (96.6)	74 (92.5)	
Dryness of tongue	Yes	16 (100)	3 (3.4)	6 (7.5)	0.001
	No	0 (0)	85 (96.6)	74 (92.5)	
Shrunken eyes	Yes	16 (100)	3 (3.4)	6 (7.5)	0.001
	No	0 (0)	85 (96.6)	74 (92.5)	
Dry skin	Yes	16 (100)	3 (3.4)	6 (7.5)	0.001
	No	0 (0)	85 (96.6)	74 (92.5)	
Tachycardia	Yes	10 (62.5)	9 (10.2)	50 (62.5)	0.001
	No	6 (37.5)	79 (89.8)	30 (37.5)	
Hypovolemic pulse	Yes	0 (0)	8 (9.1)	80 (100)	0.001
	No	16 (100)	80 (90.9)	0 (0)	
SBP <100 mmHg	Yes	0 (0)	5 (5.7)	80 (100)	0.001
	No	16 (100)	83 (94.3)	0 (0)	
JVP <3 cm	Yes	16 (100)	88 (10)	0 (0)	0.001
	No	0 (0)	0 (0)	80 (100)	
Postural hypotension	Yes	16 (100)	3 (3.4)	6 (7.5)	0.001
	No	0 (0)	85 (96.6)	74 (92.5)	
Complications					
AHF		0 (0)	0 (0)	38 (47.5)	0.001
Chronic HF		0 (0)	0 (0)	30 (37.5)	
HF with pulmonary edema		0 (0)	0 (0)	6 (7.5)	
HF with cardiogenic shock		0 (0)	0 (0)	6 (7.5)	
No HF		16 (100)	88 (100)	0 (0)	
HF with preserved EF		0 (0)	0 (0)	10 (12.5)	0.001
HF with reduced EF		0 (0)	0 (0)	70 (87.5)	
Volume status					
Hypovolemia		16 (84.2)	0 (0)	0 (0)	0.001
Normovolemia		3 (15.8)	85 (100)	0 (0)	
Hypervolemia		0 (0)	0 (0)	80 (100)	

The specificity, sensitivity, positive predictive value (PPV), and negative predictive value (NPV) of B-lines for diagnosis of HF were 88.1%, 82.7%, 80.4% and 89.6%, respectively. B-lines were >10 in 80% AHF cases, and >10 in 100% cases with chronic HF and the observed difference was significant (p<0.001). Table 3 depicts the distribution of B-lines, B-type natriuretic peptide (BNP), and N-terminal pro B-type natriuretic peptide (NT-proBNP) according to clinical features and their association with volume status and HF.

**Table 3:** Distribution of B-lines, BNP, and NT-proBNP according to clinical features and their association with volume status and heart failure Data are expressed as n (%) BNP - B-type natriuretic peptide; NT-proBNP - N-terminal pro B-type natriuretic peptide; IVC - inferior vena cava; JVP - Jugular venous pressure; HF - heart failure; EF - ejection fraction

Clinical features	B-lines		p-value	BNP		p-value	NT-proBNP		p-value
	<10	>10		<100	>100		<900	>900	
Shortness of breath	42 (43.8)	81 (92)	0.001	37 (40.7)	86 (92.5)	0.001	45 (45.5)	78 (91.80)	0.001
Orthopnea	10 (10.4)	72 (81.8)	0.001	0 (0)	82 (88.2)	0.001	8 (8.1)	74 (87.1)	0.001
Paroxysmal nocturnal dyspnea	0 (0)	57 (64.8)	0.001	0 (0)	57 (61.3)	0.001	0 (0)	57 (61.3)	0.001
S3	0 (0)	49 (55.7)	0.001	0 (0)	49 (52.7)	0.001	0 (0)	49 (52.7)	0.001
S4	20 (20.8)	22 (25)	0.50	20 (22)	22 (23.7)	0.79	20 (22)	22 (23.7)	0.79
Crepitations	0 (0)	58 (65.9)	0.001	5 (5.5)	54 (57)	0.001	5 (5.5)	54 (57)	0.001
IVC dilated and collapsible <50%	10 (10.4)	70 (79.5)	0.001	0 (0)	80 (86)	0.001	8 (8.1)	72 (84.7)	0.001
Chest X-ray infiltrated	0 (0)	58 (65.9)	0.001	5 (5.5)	53(57.1)	0.001	5 (5.5)	53(57.1)	0.001
JVP raised	10 (10.4)	70 (79.5)	0.001	0 (0)	80 (86)	0.001	8 (8.1)	72 (84.7)	0.001
Volume status									
Hypovolemia	16 (84.2)	3 (15.8)	0.001	16 (84.2)	3 (15.8)	0.001	16 (84.2)	3 (15.8)	0.001
Normovolemia	70 (82.4)	15 (17.6)		75 (88.2)	10 (11.8)		75 (88.2)	10 (11.8)	
Hypervolemia	10 (12.5)	70 (87.5)		0 (0)	80 (100)		8 (10)	72 (90)	
HF									
With reduced EF	4	70		0	70		4	76	
No HF	86	18		91	13		91	13	
With preserved EF	6	4		0	10		4	6	

**Figure 1:**
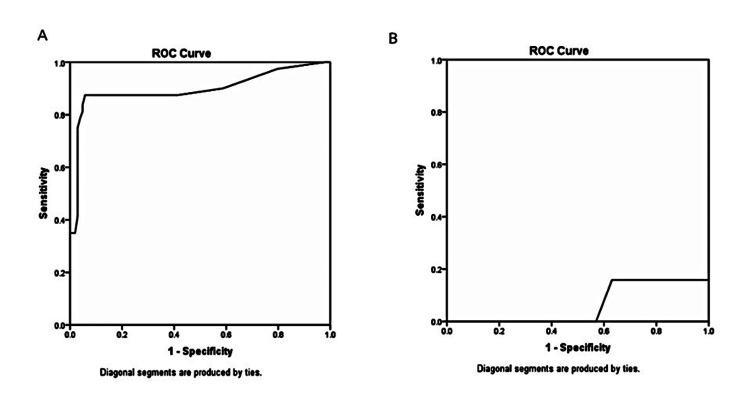
A) area under receiver operating characteristic curve of B-lines for predicting heart failure; B) area under receiver operating characteristic curve of small and collapsible inferior vena cava for predicting hypovolemia

The sensitivity, specificity, PPV, and NPV of small and collapsible IVC for diagnosing hypovolemia was 100%, 96.6%, 84.2%, and 100%, respectively. Diagnostic accuracy and Pearson's correlation coefficients of various parameters for HF with reduced and preserved EF are given in Table 4 and Table 5, respectively. Area under receiver operating characteristic (AUROC) curve for diagnosis of HF at a cut-off value of >10 for B-lines was 0.897 (0.842-0.951) (Figure 1A). AUROC for IVC size in diagnosing hypovolemia was 0.063 (0.000-0.130) (Figure 1B).

**Table 4:** Diagnostic accuracy of various parameters for heart failure with reduced and preserved ejection fraction Data are expressed as % HF - heart failure; EF - ejection fraction; BNP - B-type natriuretic peptide; NT-proBNP - N-terminal pro B-type natriuretic peptide; IVC - inferior vena cava; PPV - positive predictive value; NPV - negative predictive value

Diagnostic accuracy	HF with reduced EF	HF with preserved EF
B-lines		
Sensitivity (%)	95.6	40
Specificity (%)	82.7	82.7
PPV (%)	79.5	18.2
NPV (%)	95.6	93.5
BNP		
Sensitivity (%)	100	100
Specificity (%)	87.5	87.5
PPV (%)	84.3	47.6
NPV (%)	100	100
NT-proBNP		
Sensitivity (%)	95	60
Specificity (%)	87.5	87.5
PPV (%)	85.4	31.6
NPV (%)	95.8	95.8
Dilated and collapsible IVC		
Sensitivity (%)	100	100
Specificity (%)	100	100
PPV (%)	100	100
NPV (%)	100	100

**Table 5:** Pearson correlation coefficients of various parameters BNP - B-type natriuretic peptide; NT-proBNP - N-terminal pro B-type natriuretic peptide; IVC - inferior vena cava

Parameters	Pearson correlation coefficient	p-value
B-lines	0.74	0.001
BNP	0.867	0.001
NT-proBNP	0.868	0.001
Dilated and collapsible IVC	0.869	0.001

## Discussion

Hemodynamic assessment is crucial for diagnosing and formulating treatment strategies in MI patients. This study demonstrates that volume status can be easily assessed by the size and respiratory variation of IVC.

IVC size and volume status

In this study, small and collapsible IVC had a sensitivity and specificity of 100% and 96.6%, respectively, for hypovolemia. However, the AUROC curve for IVC size for diagnosis of hypovolemia was 0.063. Thus, it cannot accurately diagnose hypovolemia. Dilated and collapsible IVC was the only parameter with 100% diagnostic accuracy for predicting HF in patients with reduced as well as preserved EF. High right atrial pressures result in dilatation of the IVC and worsen the normal IVC collapsibility [8]. We found that dilated and collapsible IVC was highly correlated with hypervolemia (r=0.87). Ranjan et al. found that the Doppler wave inflow signal, when combined with IVC size and collapsibility, enhances the sensitivity and specificity to predict right atrial pressure to 94% and 92%, respectively [9]. De Vecchis et al. suggested that small, collapsible IVCs in echocardiography represent low right atrial pressures, whereas large, non-collapsible IVCs reflect high right atrial pressures, suggesting hypovolemia and hypervolemia, respectively [8].

Blehar et al. documented that the AUROC curve of IVC as a diagnostic criterion for congestive HF was 0.96, with a specificity and sensitivity of 84% and 92%, respectively [10]. Specificity and sensitivity reported by Kajimoto et al. for IVC collapsibility >50% were 81.1% and 83%, respectively [11]. Berthelot et al. found a Pearson correlation coefficient of 0.44-0.58 between right atrial pressure, which suggests volume status and IVC size and collapsibility, indicating that there exists only a moderate correlation between the two [12].

B-lines and volume status

In the current study, B-lines, as well as BNP and NT pro-BNP, were significantly higher in patients with hypervolemia (p<0.001). The sensitivity and specificity of B-lines in predicting HF with reduced EF were 95.6% and 82.7%, respectively. The AUROC curve for diagnosis of HF at a cut-off value of >10 for B-lines was 0.897 (range 0.842-0.951), indicating that B-lines have excellent diagnostic accuracy in HF. The sensitivity of BNP and NT pro-BNP was 100% and 95%, respectively, in the diagnosis of HF with reduced EF. In patients with HF with preserved EF, the sensitivity of BNP was 100%, and that of NT pro-BNP was 60%. Additionally, we also noted that as B-lines increased, BNP and NT pro-BNP also increased significantly (p<0.001).

Miglioranza et al. observed that B-lines had a sensitivity of 85% and specificity of 83% for the risk of decompensated HF [13]. They also found that the number of B-lines ≥15 correlates with NT pro-BNP >1000 (p<0.001). Robert et al. in their study documented sensitivities of 95% (95% confidence interval 93% to 96%) and 99% (97% to 100%) and NPV of 94% (90% to 96%) and 98% (89% to 100%), respectively, for diagnosis of AHF for BNP and NT pro-BNP [14].

This study is limited to a small number of patients in a single center. Second, jugular venous pressure estimation involves considerable interobserver variability. Last, we did not consider hypovolemia or dehydration due to other reasons for comparison of B-lines.

## Conclusions

In conclusion, volume status can be easily assessed by the size and respiratory variation of IVC. B-lines reliably indicate a cardiac origin in patients presenting with acute dyspnea, orthopnea, or paroxysmal nocturnal dyspnea. Volume status based on IVC size and B-lines revealed by echocardiography has a good predictive value (p<0.001; r=0.87 and p<0.001; r=0.74, respectively) in AMI and should be considered routinely for these patients. Dilated (>2.1 cm) and collapsible (<50%) IVC, and B-lines were significantly associated with heart failure (HF) (p<0.001; r=0.87 and p<0.001; r=0.74, respectively).
